# Evaluation of Chemical Contaminants in Conventional and Unconventional Ragusana Provola Cheese

**DOI:** 10.3390/foods11233817

**Published:** 2022-11-26

**Authors:** Luigi Liotta, Federica Litrenta, Vincenzo Lo Turco, Angela Giorgia Potortì, Vincenzo Lopreiato, Vincenzo Nava, Arianna Bionda, Giuseppa Di Bella

**Affiliations:** 1 Department of Veterinary Sciences, University of Messina, Viale Palatucci, 13, 98168 Messina, Italy; 2 Department of Biomedical, Dental and Morphological and Functional Imaging Sciences (BIOMORF), University of Messina, Viale Palatucci, 13, 98168 Messina, Italy; 3 Department of Agricultural and Environmental Sciences, Milan University, Via Celoria, 2, 20133 Milan, Italy

**Keywords:** cheese, olive cake, bisphenols, plasticizer, pesticides, food safety

## Abstract

Organic contaminants belonging to various classes (plasticizers, bisphenols, pesticides, PCBs, and PAHs,) were analyzed in samples of provola cheese produced from Friesian dairy cows fed with a conventional diet (group CTR), and an unconventional diet (group BIO) enriched with olive cake (OC). The results show that for most determined contaminants, the differences between the two diets were very slight, indicating that the contamination does not depend on the olive cake integrated in the unconventional diet. The results also indicate that the minimal contamination could result from environmental contamination or the production process. It can be concluded that unconventional provola is as safe for the consumer as conventional provola.

## 1. Introduction

Dairy products are considered, in the global economy, as one of the main markets due to their high consumption [1]. Cheese from cow’s milk is the most widely produced and consumed, compared to cheese made from the milk of other species. The cheese production is increasing today and it counts more than one millions of tons [2]. Among Italian dairy products, semi-hard pasta filata cheeses are widespread in Southern Italy, but are also highly appreciated in the rest of the peninsula. Provola pasta filata cheese produced in Ragusa (Sicily) is particularly appreciated by consumers for its sensorial characteristics such as taste and consistency, and it is a very common ingredient in many gastronomical preparations [3]. Particular attention is paid in Italy to the contamination of the dairy chain, as the dairy industry is the main food industry. There are different risks of contamination of dairy products, varying from biological to chemical compounds. The risk of chemical contamination of dairy products can result from: application of agrochemicals [4], use of veterinary drugs [5], contaminated feed and fodder [6], and the use of chemicals during production, processing and packaging [7]. Several studies have shown that more than 90% of human exposure to contaminants appears to occur through food intake [8]. Dairy products seem to be good indicators of the presence of contaminants in the food chain. Among possible contaminants, phthalates are one of the most used plasticizers in the world and belong to the group of ubiquitous environmental contaminants. High-molecular-weight phthalates, such as bis (2-ethylhexyl) phthalate (DEHP), are mainly used as plasticizers in polyvinyl chloride (PVC) products to improve the flexibility of plastics; they are not chemically bound to the polymer, so they can be released into the environment. The Environmental Protection Agency (EPA) has listed six phthalates as priority hazardous pollutants. EFSA established tolerable daily intake (TDI) of 0.01 mg/Kg bw/day, 0.5 mg/Kg bw/day, 0.05 mg/Kg bw/day, 0.15 mg/Kg bw/day, 0.15 mg/Kg bw/day for dibutyl phthalate (DBP), butyl benzyl phthalate (BBP), DEHP, diisononyl phthalate (DiNP), and di-isodecil phthalate (DiDP), respectively [9,10,11,12,13]. In 2019, EFSA set a group-TDI of 50 µg/Kg bw/day for DBP, BBP, DEHP, and DiNP [14]. Due to their lipophilic nature, phthalates are mainly found in high-fat dairy products [15,16] and can accumulate in feedstuffs and ruminant tissues, in fatty muscles, and pass from the animal’s digestive tract to milk and, consequently, to humans [17]. Many studies have been conducted on the migration of phthalates from PVC pipes to milking machines used on farms [18,19,20].

Great attention from the scientific community is paid to bisphenols (BPs), which are used in the production of polycarbonate plastics and epoxy resins. Bisphenols can enter the dairy chain through animal feed, production machinery, environmental contamination, and packaging materials [21]. Bisphenols can be accumulated in the adipose tissue of the animal, secreted in milk fat, and stored in fatty dairy products [22]. EFSA established a tolerable daily intake (TDI) of 0.04 ng/kg body weight/day for 4,4′-(propan-2,2-diyl) diphenol (BPA) [23], and the European Union setting for it a specific migration limit (SML) of 0.05 mg/kg in food (Regulation (EU) No 10/2011). Due to strict regulations, structural analogues of bisphenol A are being used as substitutes for it, and they are gradually entering the dairy chain [24]. Regulation (EU) No 10/2011 establishes that 4,4′-sulfonyldiphenol (BPS) is authorized to be used as a monomer in plastic food contact materials with a specific migration limit (SML) of 0.05 mg/kg food. There is not a TDI value fixed for BPS.

Concerning pesticides, polychlorinated biphenyls (PCBs) and polycyclic aromatic hydrocarbons (PAHs) it is known that the risk of transmission through the food chain and through milk is high [25]. These contaminants are highly lipophilic and resistant to degradation [26], and can be biomagnified through the food chain. In addition, their highly toxic nature can cause adverse effects on both human health and the environment [27]. Some of these have been banned in many countries because of their persistence in the environment and their potential adverse effect on human health, according to the Stockholm Convention adopted in 2001 and entered into force in 2004. Many of these classes of contaminants mentioned have been designated as endocrine disruptors [28] and, consequently, particularly dangerous due to their carcinogenic, mutagenic, and teratogenic effects. 

The aim of this study was to evaluate the food safety of Provola Ragusana cheese by determination of plasticizers, bisphenols, pesticides, PCBs, and PAHs residues, also taking into account the different types of products obtained according to the QS (Safe Quality) disciplinary approved by Sicilian Region Government. The QS mark foresees the use of de-stoned olive cake in cattle feed as a tool for the sustainability of the agri-food chain.

## 2. Materials and Methods

### 2.1. Chemicals and Reagents

All solvents and reagents were of analytical grade; acetonitrile, water and n-hexane were purchased from Fluka Sigma-Aldrich S.r.l. (Milan, Italy). Analytical standards of phthalate acid esters (PAEs) and non-phthalate plasticizers (NPPs) (certified purity ≥ 96%) were provided by Supelco (Bellefonte, PA, USA). Internal standards (IS), dibutyl phthalate-d4 (DBP-d4) and bis(2-ethylhexyl)phthalate-d4 (DEHP-d4) in nonane (100 μg/mL each) were purchased from Cambridge Isotope Laboratories Inc. (Andover, MA, USA); All the PAEs and NPPs standard were dissolved in hexane to prepare a mixed stock standard solution (10 μg/mL). Analytical standards of bisphenols were purchased from Sigma-Aldrich (Steinheim, Germany). ^13^C_12_-BPA was obtained from Cambridge Isotope Laboratories, Inc. (Andover, MA, USA). The standard stock solutions (1 mg/mL) of all target analytes and internal standard (^13^C_12_-BPA) were prepared in acetonitrile. Standards of 109 pesticides, 18 PCBs, 13 PAHs were purchased from Aldrich Chemical (Chicago, IL, USA), Fluka Analytical (Milan, Italy) and Dr. Ehrenstorfer (Augsburg, Germany). All details are showed in Table S1. Stock standard solutions of individual standards were prepared by dissolving pure standard in n-hexane (1000 mg/L). A standard solution of bromophos-methyl, used as internal standard (IS), was prepared at 50 ng/mL in n-hexane. The Quick, Easy, Cheap, Effective, Rugged, and Safe (QuEChERS) Q-sep extraction kit (4 g MgSO_4_ and 1 g NaCl and MgSO_4_ 6 g + NaOAc 1.5 g) were purchased from Agilent Technologies Italia S.p.A. (Milan, Italy).

### 2.2. Samples Collection

Provola samples were collected from March to July 2021 at a commercial cheesemaking located in Ragusa (Sicily, Italy). Provola cheese was produced from milk of Friesian cows subjected to two different feeding systems: a conventional diet (CTR) and an unconventional diet (BIO) similar in composition as showed in Table S2; the only difference was pitted olive cake (OC) that was added in BIO diet at 8% level on Dray Matter (DM).

**Figure 1:**
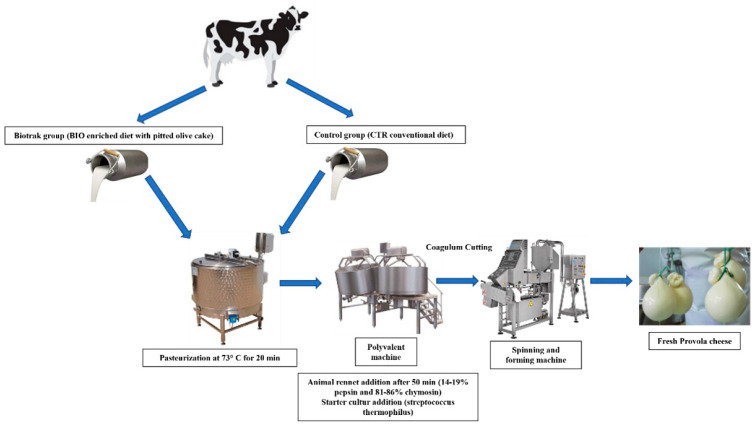
Production process diagram of provola samples.

Monthly, 400 L of milk from each group were cheesed into 80 provolas according to the production diagram shown in Figure 1. Four representative samples of provolas obtained were analyzed. Each representative sample was obtained from five Provolas randomly selected from 20 provalas. In addition, the dried and pitted olive cake used as a supplement in the BIO diet was stored at the beginning of the trial and then sampled and analyzed monthly.

### 2.3. Extraction of Samples

The extraction of the different classes of contaminants was conducted using different QuEChERS methods according to the matrix type. Briefly, the procedure followed consisted of a first step (extraction) in which the homogenized sample was extracted with acetonitrile (ACN), followed by the addition of salts such as anhydrous magnesium sulphate (MgSO_4_), sodium chloride (NaCl), and sodium acetate (NaOAc). In a second step (clean-up), an aliquot of the upper layer was cleaned with primary and secondary amine (PSA), MgSO_4_ and C18. Table 1 and Table 2 show the details of analytical methods.

### 2.4. Instrumentation and Analytical Conditions

#### 2.4.1. GC-MS/MS Analysis

The simultaneous analysis of 109 pesticides, 18 PCBs, 13 PAHs belonging to different classes and of 10 PAEs and 8 NPPs were performed using a Shimadzu TQ8030 HRGC-MS/MS (Shimadzu Italia, Milan, Italy), equipped with a Shimadzu AOC-20s autosampler and a Supelco SLB-5MS capillary column (5% polydiphenylsiloxane, 95% polydimethylsiloxane) 30 m × 0.25 mm i.d., 0.25 µm film thickness, operating in electronic ionization (EI) mode. For pesticides PCBs and PAHs the instrumental conditions were: He carrier gas at flow rate 0.5 mL/min; Ar collision gas; column temperature: 60 °C (1 min), 15 °C/min up to 150 °C, 10 °C/min up to 270 °C, 2 °C/min up to the final temperature of 300 °C (2 min); injector temperature: 250 °C; ion source temperature (EI, 70 eV): 230 °C; transfer line temperature: 290 °C; injection volume was 1 µL, in split-less mode (1 min) and finally with a split ratio of 1:10. All quantitative and qualitative data were acquired in multiple reaction monitoring (MRM) mode according to previous studies [33,34]. All details are reported in Tables S3 and S4. For PAEs and NPPs the instrumental conditions were: He carrier gas at flow rate 0.68 mL/min; column temperature was initially varied from 60 °C to 190 °C, then 8 °C/min up to 240 °C (5 min), 8 °C/min up to the final temperature of 315 °C (5 min); injector temperature: 250 °C; ion source temperature (EI, 70 eV): 200 °C; injection volume was 1 µL, in split-less mode (1 min), and finally with a split ratio of 1:15. The assay was performed in single-ion monitoring (SIM) mode. SIM was done using three characteristic mass fragments: the first being the target ion (T) and the last two the qualifying ions (Q1 and Q2) for each target analyte [35]. All details are reported in Table S5.

#### 2.4.2. HPLC-MS/MS Analysis

The determination of 9 BPs were performing using HPLC system (Shimatzu, Kyoto, Japan), consisting of an LC-20ADXR binary pump, a SIL-20AXR autosampler, and temperature-controlled column operator. The detector was a LCMS-8040 triple quadrupole mass spectrometer with Electrospray ionisation (ESI) source. Labsolution software was used for data control and analysis. Chromatographic separation was performed on an Agilent Zorbax SB-C18 column (5microm 4.6 × 250 mm). The flow rate was 0.7 mL/min. Mobile phases A and B were ultrapure water for Hplc-MS and acetonitrile, respectively. The following linear gradient was used: 0 min, 20%B; 7 min, 40%B; 25 min, 90%B; 35 min, 20%B. The injection volume was 20 µL and the column temperature was set at 40 °C. The MS was operated in negative ESI mode under the following specific conditions: nebulizer gas flow 3.0 L/min, nebulizer gas pressure 770 KPa, drying gas flow 15.0 L/min, DL temperature 250 °C, CID gas 230 KPa. The dwell time was set to 500 ms. All quantitative and qualitative data in this study were acquired in multiple reaction monitoring mode (MRM). All details are reported in Table S6.

### 2.5. Statistical Analysis

The statistical analyses were carried out using JMP^®^ 16 software (SAS Institute Inc., Cary, NC, USA, 1989-2021). For each parameter, descriptive statistics was generated and reported as mean ± standard deviation (SD). A two-way analysis of variance (ANOVA) with interaction was used applied to all the measured parameters and the Tukey-Kramer post-hoc test was used to identify the different levels when the model factors resulted significant; the included factors are reported below:

Yijk = m + Di + Mj + (DM)ij + eijk(1)

where m is the mean, Di is the diet (CTR vs. BIO), Mj is the month in which the cheese was produced (from March 2021 to July 2021), (DM)ij is the interaction between the diet and the month, and eijk is the random residual. A principal component analysis (PCA) was performed using all the measured contaminants.

## 3. Results 

### 3.1. PAEs, NPPs and BPs in Ragusana Provolas

The concentrations of bisphenols and plasticizers detected in provola samples together with the significance of each factor for each parameter analyzed are shown in Table 3. Bisphenols 4,4′-methylenediphenol (BPF), 1,1-Bis(4-hydroxyphenyl) ethane (BPE), 1,1-Bis(4-hydroxyphenyl)-1-phenyl-ethane (BPAP), 1,1-Bis(4-hydroxyphenyl)-cyclohexane (BPZ), 1,4-Bis(2-(4-hydroxyphenyl)-2-propyl)benzene (BPP), 4-[2-(4-hydroxyphenyl) butan-2-yl] phenol (BPB), BPA and plasticizers butyl benzyl phthalate (BBP), diphenyl phthalate (DPhP), dicyclohexyl phthalate (DeHexP), diheptyl phthalate (DHepP), diethyl adipate (DEA), benzyl benzoate (BB), dibutyl adipate (DBA), and diisobutyl adipate (DiBA) were not detected in any samples. In provola samples, the concentrations of BPAF and BPS varied from 2.06 to 2.84 μg/Kg and from 1.69 to 2.52 μg/Kg, respectively. Significant differences are observed between BIO and CTR for the months of March and June. In July only, significant differences are observed for BIO provolas. While a significant increase is observed for CTR from March to May and from May onward, however, the differences are not significant. Significant seasonal variability is observed for both BIO and CTR in the phthalates detected. Significantly lower phthalate values were registered in April and June. No significant differences were observed between BIO and CTR in March and July. Significant differences by sampling period were always shown for dimethyl adipate (DMA) and di(2-ethylhexyl) adipate (DEHA) in provola, with significantly lower values in April and May. Furthermore, no significant differences were shown for DMA and DEHA between BIO and CTR in months. Di(2-ethylhexyl) terephthalate (DEHT) was found only in the provolas with values in the range of 51.43 μg/Kg to 124.08 μg/Kg. In July, DEHT was significantly higher for both BIO and CTR and the concentrations were significantly different from each other. 

### 3.2. Pesticides, PAHs and PCBs in Ragusana Provolas

The concentrations of pesticides and PAHs detected in provolas together with the significance of each factor for each parameter analyzed are shown in Table 4. PCBs values in all provola samples were below the limit of quantification (LOQ). In provolas were detected anthracene and fluorene with concentrations in the ranges of 0.39–0.45 μg/Kg and 0.26–0.31 μg/Kg, respectively. No significant difference was observed for CTR in months, while for BIO higher values were observed in April and lower values in June. Among the pesticides sought, only eight residues were determined (Carbaryl, t-Fluvalinate, Pyriproxyfen, Dieldrin, Phosalone, Clorpirifos, Tebuconazole, and Fenchlorfos). Carbaryl and t-Fluvalinate had the highest concentrations. No differences were observed between the CTR and BIO samples.

### 3.3. PAEs, NPPs, BPs, Pesticides, PCBs and PAHs in Olive Cake

Monthly, from the stored olive cake, a sample was taken and subjected to analysis. The results were not significantly different over the months. 

The obtained average concentrations for each contaminant found in olive cake are shown in Table 5. BPA, BPZ, BPB, and BPS in olive cake samples were determined. Except for BPS, which content is ubiquitous in all samples, the other bisphenols were not detected in provolas. BPB was the most abundant, with a concentration of 20.99 ± 0.04 μg/Kg, followed by BPA 14.50 ± 0.36 μg/Kg, and BPZ 13.12 ± 0.05 μg/Kg. The same plasticizers determined in olive cakes were also determined in both BIO and CTR cheese samples. In the latter, sometimes even with significantly higher values. Additionally, in all provola samples, residue of diethyl phthalate (DEP), diisobutyl phthalate (DiBP), and dipropyl phthalate (DPrP) were also found. 

Pesticide residues determined in the olive cake were different from those in provolas, except for carbaryl and tebuconazole residues, that were also determined in all provolas samples. In olive cake, of all the PAHs analyzed, only anthracene and fluorene residues were found, as well as in provolas. 

### 3.4. PCA Analysis

**Figure 2:**
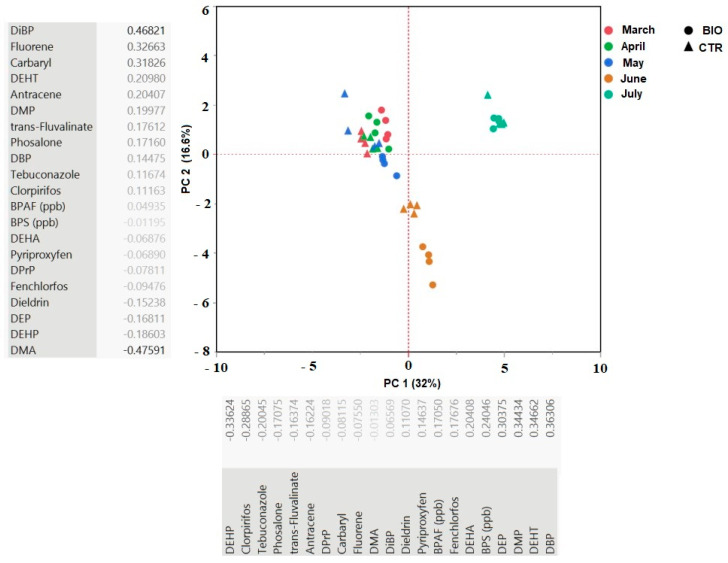
Scores plot for the first two principal components. Each month is represented by a different color; BIO and CTR samples are represented by circles and triangles, respectively.

According to Kaiser Criterion, only those PCs with eigenvalues greater than unity were retained. The six extracted principal component (eigenvalues 6.7212, 3.4867, 2.3888, 1.8118, 1.5805 and 1.4958, respectively) explains up to 83.260% of the total variance (32.006%, 16.603%, 11.375%, 8.628%, 7.526% and 7.123%, respectively). The first component shows the highest positive correlations with DBP (0.36), DEHT (0.35), DMP (0.34) and DEP (0.30), while negative correlations can be observed for DEHP (−0.34) and Chlorpyrifos (−0.29); DiBP and to a lesser extent fluorene and carbaryl have positive correlation with the second component (0.47, 0.33 and 0.32, respectively) while DMA had negative correlation (−0.48). In the PCA, samples tended to group according to the month factor (Figure 2). In particular, the first principal component, which explained 32% of the overall variability, clearly distinguished the provolas produced in June and July. In particular, the July samples are characterized by higher values of DBP, DEHT, dimethyl phthalate (DMP), for both BIO and CTR, whereas the June samples are characterized by significantly higher values of DMA and lower values of DiBP. As expected, the diets were not clearly distinguished; however, BIO samples were plotted at PC 1 value more positive than the CTR samples of the same month in most of the cases. The greatest difference between the two diets was seen in June and May; in May, we could also observe a greater variability with respect to the other months. 

## 4. Discussion

### 4.1. Chemical Contaminants in Provola Cheese

In the present study, levels of BPAF and BPS residues were detected in samples of provola. Information on contamination levels of BPA analogues in dairy products is still limited. Furthermore, with the exception of BPS, all bisphenols found in olive cake are not present in provola, suggesting that the contribution of bisphenols in provola is not dependent on dietary supplementation of olive cake in the animal’s diet. Bisphenols present in OC were probably metabolized and excreted by the animal through urine in accordance with Sonker et. al [36]. The levels of ubiquitous contamination of provola cheese suggest that BPAF and BPS could result from contact with the plastic material in the machinery during the production process, and heat treatments during milk processing could have promoted the leaching of BPs into the cheese [37,38]. Furthermore, another likely source could be environmental contamination during the farming phase [39]. PAEs and NPPs in provolas and olive cake were determined. In provola samples from April and June, lower phthalate values were found, probably due to seasonal variations. These results are in accordance with a previous study [20] in which PAEs levels appeared to vary seasonally. The same trend was observed for DMA and DEHA. In accordance with previous studies, there are several sources of possible migration of phthalates and adipates during the entire process; it is known, for example, that heat accelerates the migration of phthalates [40,41], thus facilitated by pasteurization, or through contact with PVC tubes or plastic seals in the machinery used during processing [42]. DEHT was found in provolas; higher values were found in July for both BIO and CTR, which is probably due to the high seasonal temperature [40]. Furthermore, some studies (https://www.kleanupkraft.org/Phthalates-Farm-Equipment.pdf (accessed on 30 October 2022)) show that DEHT, due to its fewer hazardous properties, is used as a substitute for DEHP in the machinery used in dairy production. This justifies the presence of DEHT in provola as process-contamination. The results showed that BIO samples had similar or lower levels for almost all the contaminants.

### 4.2. Dietary Exposure to Plasticisers and Bisphenols through Provola Cheese 

The presence of BPA analogues and plasticizers such as DEP, DBP, DEHP, and DEHA in the analyzed samples necessitates the assessment of their dietary exposure. The present study is the first to investigate human exposure to bisphenols and plasticizers through the consumption of provola cheese.

Based on the results obtained from the provola samples, the estimated dietary intake (EDI) of these compounds was calculated using the following equation [43]:

EDI = C·D/BW(2)

where C (μg/Kg) is the average measured concentration of the analytes in the provola samples. D (Kg/day) is the daily consumption dose (0.045 Kg/day) according to Crupi et al. [44] and recommended by Food and Agriculture Organization of the United Nations (FAOSTAT) [45], and BW (kg) is the average adult body weight (60 kg).

For the health risk assessment, the hazard quotient (HQ) for each plasticizers detected was calculated using the following equation [46]:

HQ = EDI/TDI(3)

HQ values of less than 1 are considered safe, while values higher than 1 indicate that there is a possibility of inducing adverse health effects in the consumer [46]. EFSA, in 2005, established a TDI of 0.01 mg/kg BW/day and 0.05 mg/kg BW/day for DBP and DEHP, respectively. The TDI for DEP was set in 2003 by the World Health Organization (WHO), which proposed a TDI of 5.00 mg/kg bw/day [47]. The SCF has confirmed for DEHA a TDI of 0.30 mg/kg bw/day in 2000 [48].

Due to the unavailability of TDI values for BPA analogues, it was not possible to calculate the hazard quotient for analogues. The European Food Safety Authority [23] established a TDI of 0.04 ng/kg BW/day for BPA alone. 

The main results of EDI and HQ calculation are reported in Table 6. The results for samples categorized by diet and period are reported in Table S7. In this study, the average EDI in the CTR samples was 1.9 × 10^−03^ ± 2.7 × 10^−04^ μg/Kg_bw_/day and 1.7 × 10^−03^ ± 2.4 × 10^−04^ μg/Kg_bw_/day for BPAF and BPS, respectively. In BIO samples it was 1.9 × 10^−03^ ± 1.0 × 10^−04^ μg/Kg and 1.6 × 10^−03^ ± 1.4 × 10^−04^ μg/Kg for BPAF and BPS, respectively. These results indicate that the estimated dietary intake is similar in the two diets. 

For DEP, DBP, DEHA, and DEHP both EDI and HQ can be calculated. HQ values are well below 1 for all determined plasticizers, suggesting that there is no hazard for the consumer linked with these contaminants in analyzed provola samples. 

## 5. Conclusions

In this study, the presence of plasticizers, bisphenols, pesticides, PCBs, and PAHs was evaluated in provola from dairy cows fed a conventional diet and an unconventional diet enriched with dried olive cake. The same contaminants were evaluated in olive cake samples. 

The results highlight that no variation was observed throughout the months for olive cake. In provola samples, only minimal differences were noted, indicating that the contamination does not depend on the olive cake supplemented in the BIO diet. Therefore, in this case, the source of contamination is not attributable to olive cake, but probably depends on the production process or ubiquitous environmental contamination. It can be concluded that unconventional provola is as safe for the consumer as conventional provola, thus representing a good animal husbandry strategy of sustainability.

## Figures and Tables

**Table 1:** Analytical methods for PAEs, NPPs and BPs.

Matrix	Analytes	Extraction		Instrument	Reference
		Solvent and Salts	Clean-Up		
Provola	10 PAEs 8 NPPs	(1) 5 g of homogenized sample with 80.0 µL of the SI (20 µg/mL) was extracted with 10.0 mL of ACN; (2) 3 g NaCl was added and centrifuged for 5 min.	(3) 2.0 mL of the top layer of ACN was transferred into QuEChERS d-SPE (300 mg MgSO_4_, 50 mg PSA, 50 mg C18) and shaken vigorously for 5 min, then centrifuged for 5 min; (4) 1.0 mL of the upper layer was dried with N_2_ flow at 40 °C, the residual was dissolved in 1.0 mL n-hexane.	GC-MS/MS	Fan et al., 2019 [29]
Provola	9 BPs	(1) 5 g of homogenized sample spiked with SI was extracted with 5.0 mL of ACN; (2) 2 g NaCl and 1 g MgSO_4_ was added, shaken for 5 min and centrifuged for 5 min.	(3) 2.0 mL of the top layer of ACN was transferred into QuEChERS d-SPE (0.25 mg MgSO_4_, 0.1 mg PSA, 0.1 mg C18), vortexed for 30 s, and centrifuged for 5 min; (4) 1.0 mL of the upper layer was filtered through a PTFE Millipore filter (0.22 µm).	HPLC-MS/MS	Xiong et al., 2018 [30]
Olive cake	10 PAEs 8 NPPs	(1) 3 g of dried olive cake with 80.0 µL of the SI (20 µg/mL) was extracted with 7 mL of H_2_O and 10 mL of ACN; (2) 4 g MgSO_4_ and 1 g NaCl were added and vortexed for 1 min; the extract was centrifuged for 10 min.	(3) 5.0 mL of the top layer of ACN was transferred into QuEChERS d-SPE (750 mg MgSO_4_, 250 mg PSA and 250 mg C18), shaken vigorously for 5 min, and centrifuged. (4) 1.0 mL of supernatant was recovered, dry-carried and reconstituted with 1 mL of n-hexane.	GC-MS/MS	Santana-Mayor et al., 2019 [31]
Olive cake	9 BPs	(1) 3 g of homogenized sample spiked with SI was extracted with 5.0 mL of ACN; (2) 2 g NaCl and 1 g MgSO_4_ was added, shaken for 5 min and centrifuged for 5 min.	(3) 2.0 mL of the top layer of ACN was transferred into QuEChERS d-SPE (0.25 mg MgSO_4_, 0.1 mg PSA, 0.1 mg C18), vortexed for 30 s, and centrifuged for 5 min; (4) 1.0 mL of the upper layer was filtered through a PTFE Millipore filter (0.22 µm).	HPLC-MS/MS	Xiong et al., 2018 [30]

PAE, phthalate acid esters; NPP, non-phthalate plasticizers; BP, bisphenols; HPLC-MS/MS, high performance liquid chromatography- tandem mass spectrometry; GC-MS/MS, Gas chromatography-tandem mass spectrometry.

**Table 2:** Analytical methods for Pesticides, PCBs and PAHs.

Matrix	Analytes	Extraction		Instrumental	Reference
		Solvent and Salts	Clean-Up		
Provola	106 Pesticides 18 PCB 13 PAHs	(1) 15 g of homogenized sample with bromophos-methyl (50 ng/mL) was extracted with 20 mL ACN for 5 min; (2) 6 g MgSO_4_ and 1.5 g NaOAc were added and vortexed for 1 min; the extract was centrifuged for 15 min.	(3) 5 mL of the top layer of ACN was transferred into QuEChERS d-SPE (1200 mg MgSO_4_, 400 mg PSA, 400 mg C18) and shaken vigorously for 5 min, then centrifuged for 5 min; (4) 1 mL of supernatant was recovered, brought to dryness and reconstituted with 1 mL of n-hexane.	GC-MS/MS	Golge et al., 2018 [32]
Olive cake	106 Pesticides 18 PCBs 13 PAHs	(1) 3 g of dried olive cake with bromophos-methyl (50 ng/mL) was extracted with 7 mL of H_2_O and 10 mL of ACN; (2) 4 g MgSO_4_ and 1 g NaCl were added and vortexed for 1 min; the extract was centrifuged for 10 min.	(3) 5 mL of the top layer of ACN was transferred into QuEChERS d-SPE (750 mg MgSO_4_, 250 mg PSA and 250 mg C18), shaken vigorously for 5 min, and centrifuged. (4) 1 mL of supernatant was recovered, brought to dryness and reconstituted with 1 mL of n-hexane.	GC-MS/MS	Santana-Mayor et al., 2019 [31]

PCB, polychlorinated biphenyls; PAH, polycyclic aromatic hydrocarbons; GC-MS/MS, Gas chromatography-tandem mass spectrometry.

**Table 3:** Bisphenols and plasticizers (µg/Kg) in Provola samples.

		BPAF	BPS	DMP	DEP	DPrP	DiBP	DBP	DEHP	DMA	DEHA	DEHT
CTR												
	March	2.06 ± 0.07 ^h^	1.69 ± 0.05 ^c^	4.26 ± 0.02 ^d^	6.07 ± 0.05 ^b^	3.22 ± 0.07 ^ab^	16.08 ± 0.03 ^c^	6.10 ± 0.01 ^f^	21.05 ± 0.04 ^a^	4.60 ± 0.07 ^h^	98.26 ± 0.03 ^b^	51.45 ± 0.13 ^g^
	April	2.24 ± 0.02 ^g^	2.20 ± 0.07 ^ab^	4.04 ± 0.02 ^h^	5.38 ± 0.02 ^d^	2.67 ± 0.02 ^f^	15.08 ± 0.01 ^d^	5.76 ± 0.02 ^g^	19.04 ± 0.02 ^e^	5.17 ± 0.02 ^d^	50.29 ± 0.05 ^g^	60.07 ± 0.03 ^c^
	May	2.84 ± 0.02 ^a^	2.19 ± 0.61 ^ab^	4.20 ± 0.02 ^e^	5.23 ± 0.01 ^de^	3.31 ± 0.01 ^a^	16.02 ± 0.03 ^c^	6.22 ± 0.02 ^f^	20.21 ± 0.02 ^b^	4.89 ± 0.01 ^f^	46.50 ± 0.99 ^h^	52.30 ± 0.03 ^f^
	June	2.72 ± 0.12 ^abc^	2.46 ± 0.04 ^ab^	4.33 ± 0.02 ^c^	7.02 ± 0.02 ^a^	3.01 ± 0.02 ^cd^	12.19 ± 0.02 ^e^	6.53 ± 0.03 ^c^	20.30 ± 0.04 ^b^	7.03 ± 0.04 ^b^	90.28 ± 0.02 ^d^	54.10 ± 0.01 ^d^
	July	2.80 ± 0.02 ^ab^	2.52 ± 0.08 ^a^	5.02 ± 0.01 ^b^	7.11 ± 0.03 ^a^	2.85 ± 0.04 ^e^	17.41 ± 0.2 ^a^	9.05 ± 0.01 ^a^	14.20 ± 0.02 ^g^	4.75 ± 0.02 ^g^	95.06 ± 0.04 ^c^	124.08 ± 0.07 ^a^
BIO												
	March	2.32 ± 0.01 ^fg^	2.03 ± 0.03 ^bc^	4.26 ± 0.01 ^d^	6.10 ± 0.01 ^b^	3.16 ± 0.16 ^b^	16.10 ± 0.04 ^c^	6.23 ± 0.02 ^e^	21.10 ± 0.07 ^a^	4.58 ± 0.03 ^h^	99.06 ± 0.05 ^a^	51.43 ± 0.10 ^g^
	April	2.54 ± 0.07 ^de^	2.05 ± 0.04 ^abc^	4.17 ± 0.01 ^ef^	5.72 ± 0.21 ^c^	2.60 ± 0.06 ^f^	15.12 ± 0.03 ^d^	5.82 ± 0.09 ^g^	19.16 ± 0.06 ^e^	5.25 ± 0.03 ^c^	51.21 ± 0.04 ^f^	59.96 ± 0.06 ^c^
	May	2.59 ± 0.03 ^cd^	2.00 ± 0.01 ^bc^	4.11 ± 0.02 ^g^	5.17 ± 0.02 ^e^	3.17 ± 0.01 ^b^	16.07 ± 0.06 ^c^	6.16 ± 0.01 ^ef^	20.05 ± 0.10 ^c^	4.98 ± 0.02 ^e^	45.19 ± 0.03 ^i^	53.00 ± 0.02 ^e^
	June	2.41 ± 0.03 ^ef^	2.17 ± 0.05 ^abc^	4.13 ± 0.03 ^fg^	7.03 ± 0.02 ^a^	3.06 ± 0.02 ^c^	12.09 ± 0.05 ^f^	6.41 ± 0.01 ^d^	19.86 ± 0.05 ^d^	7.12 ± 0.02 ^a^	89.79 ± 0.16 ^e^	54.08 ± 0.02 ^d^
	July	2.66 ± 0.02 ^bcd^	2.44 ± 0.02 ^ab^	5.11 ± 0.01 ^a^	7.06 ± 0.01 ^a^	2.93 ± 0.02 ^de^	16.61 ± 0.01 ^b^	8.96 ± 0.04 ^b^	15.19 ± 0.07 ^f^	4.30 ± 0.02 ^i^	95.10 ± 0.02 ^c^	119.70 ± 0.07 ^b^
*p*-value												
	Diet	0.2093	0.2666	**0.0371 I**	**0.0231 II**	**0.0191 I**	**<0.0001 I**	0.1379	**<0.0001 II**	**0.0006 I**	0.87	**<0.0001 I**
	Month	**<0.0001**	**<0.0001**	**<0.0001**	**<0.0001**	**<0.0001**	**<0.0001**	**<0.0001**	**<0.0001**	**<0.0001**	**<0.0001**	**<0.0001**
	Diet × Month	**<0.0001**	**0.0366**	**<0.0001**	**<0.0001**	**<0.0001**	**<0.0001**	**<0.0001**	**<0.0001**	**<0.0001**	**<0.0001**	**<0.0001**

Different letters represent significantly different mean values through months (*p* < 0.05). Bold value represent significantly different mean value between the two diets (*p* < 0.05). Next to the significant *p*-values for the diet is a II if the values are greater for the biotrak (BIO) diet and I if they are greater in the conventional diet (CTR).

**Table 4:** PAHs and pesticides content (µg/kg) in Provola samples.

		Antracene	Fluorene	Carbaryl	t-Fluvalinate	Pyriproxyfen	Dieldrin	Phosalone	Clorpirifos	Tebuconazole	Fenchlorfos
CTR											
	March	0.43 ± 0.02	0.30 ± 0.01 ^ab^	10.32 ± 0.11 ^ab^	5.19 ± 0.02 ^bcde^	3.43 ± 0.03 ^e^	0.52 ± 0.01 ^a^	0.34 ± 0.04 ^a^	0.31 ± 0.01 ^a^	0.12 ± 0.01 ^abc^	0.02 ± 0.01 ^d^
	April	0.42 ± 0.02	0.29 ± 0.01 ^ab^	10.40 ± 0.01 ^ab^	5.29 ± 0.08 ^abc^	3.95 ± 0.10 ^d^	0.43 ± 0.07 ^b^	0.36 ± 0.10 ^a^	0.29 ± 0.07 ^a^	0.10 ± 0.01 ^bc^	0.02 ± 0.01 ^d^
	May	0.44 ± 0.06	0.28 ± 0.02 ^abc^	10.35 ± 0.06 ^ab^	5.40 ± 0.10 ^a^	3.75 ± 0.32 ^de^	0.47 ± 0.04 ^ab^	0.41 ± 0.08 ^a^	0.30 ± 0.02 ^a^	0.13 ± 0.01 ^ab^	0.03 ± 0.01 ^cd^
	June	0.42 ± 0.03	0.29 ± 0.01 ^abc^	10.24 ± 0.05 ^ab^	5.29 ± 0.09 ^abcd^	4.06 ± 0.06 ^cd^	0.46 ± 0.05 ^ab^	0.34 ± 0.05 ^a^	0.22 ± 0.02 ^cd^	0.12 ± 0.01 ^abc^	0.04 ± 0.01 ^cd^
	July	0.41 ± 0.01	0.29 ± 0.01 ^abc^	10.28 ± 0.03 ^ab^	5.15 ± 0.07 ^de^	4.14 ± 0.05 ^bcd^	0.50 ± 0.02 ^ab^	0.34 ± 0.06 ^a^	0.20 ± 0.02 ^d^	0.09 ± 0.02 ^c^	0.05 ± 0.01 ^abc^
BIO											
	March	0.41 ± 0.01	0.30 ± 0.01 ^ab^	10.66 ± 0.46 ^a^	5.33 ± 0.02 ^ab^	4.67 ± 0.20 ^a^	0.27 ± 0.01 ^c^	0.42 ± 0.01 ^a^	0.23 ± 0.03 ^c^	0.12 ± 0.01 ^abc^	0.07 ± 0.01 ^a^
	April	0.45 ± 0.04	0.31 ± 0.02 ^a^	10.29 ± 0.01 ^ab^	5.14 ± 0.04 ^e^	4.57 ± 0.14 ^ab^	0.29 ± 0.02 ^c^	0.40 ± 0.01 ^a^	0.26 ± 0.01 ^bc^	0.13 ± 0.02 ^a^	0.04 ± 0.02 ^bc^
	May	0.43 ± 0.01	0.28 ± 0.01 ^bc^	10.28 ± 0.05 ^ab^	5.10 ± 0.05 ^e^	4.40 ± 0.40 ^abc^	0.52 ± 0.02 ^a^	0.33 ± 0.02 ^a^	0.27 ± 0.01 ^b^	0.11 ± 0.01 ^abc^	0.06 ± 0.01 ^ab^
	June	0.39 ± 0.01	0.26 ± 0.01 ^c^	9.90 ± 0.48 ^b^	5.07 ± 0.02 ^e^	4.49 ± 0.04 ^abc^	0.51 ± 0.01 ^a^	0.33 ± 0.01 ^a^	0.21 ± 0.01 ^cd^	0.10 ± 0.01 ^c^	0.07 ± 0.01 ^a^
	July	0.40 ± 0.01	0.29 ± 0.02 ^ab^	10.30 ± 0.01 ^ab^	5.14 ± 0.05 ^de^	4.59 ± 0.07 ^a^	0.48 ± 0.04 ^ab^	0.31 ± 0.01 ^a^	0.20 ± 0.01 ^d^	0.10 ± 0.01 ^abc^	0.07 ± 0.01 ^a^
*p*-value											
	Diet	0.6252	0.5969	0.6823	**<0.0001 I**	**<0.0001 II**	**<0.0001 I**	0.8715	**0.001 I**	1	**<0.0001 II**
	Month	0.0605	**0.0001**	**0.0107**	**0.0033**	**0.0064**	**<0.0001**	0.0647	**<0.0001**	**0.013**	**<0.0001**
	Diet × Month	0.2138	**0.0175**	**0.0499**	**<0.0001**	**0.0008**	**<0.0001**	**0.0322**	0.0571	**0.0006**	**0.0459**

Different letters represent significantly different mean values through months (*p* < 0.05). Bold value represent significantly different mean value between the two diets (*p* < 0.05). Next to the significant *p*-values for the diet is a II if the values are greater for the biotrak (BIO) diet and I if they are greater in the conventional diet (CTR).

**Table 5:** Bisphenols, plasticizers, PAHs and pesticides average content (µg/kg) in olive cake (*n* = 5).

	Value
**Bisphenols**	
BPA	14.50 ± 0.36
BPZ	13.12 ± 0.05
BPB	20.99 ± 0.04
BPS	3.27 ± 0.15
**Plasticizers**	
DMP	9.20 ± 0.01
DBP	11.12 ± 0.03
DEHP	12.91 ± 0.42
DMA	7.64 ± 0.05
DEHA	45.52 ± 0.10
DEHT	15.38 ± 0.03
**PAHs**	
Anthracene	0.66 ± 0.02
Fluorene	0.92 ± 0.02
**Pesticides**	
Carbaryl	0.50 ± 0.04
Tebuconazole	0.33 ± 0.02
Carbophenothion	3.12 ± 0.04
Azinphos-ethyl	0.75 ± 0.03
Dicofol	0.23 ± 0.02
Fenarimol	0.62 ± 0.02
Furathiocarb	5.43 ± 0.14
Acenaphthylene	0.49 ± 0.02
Fhosmet	0.67 ± 0.02
Omethoate	20.84 ± 0.31
Endosulfan	0.95 ± 0.07
Quinalphos	0.93 ± 0.03

**Table 6:** Estimated Daily Intake (mean ± standard deviation, µg/Kg_bw_/day), Hazard Index and Tolerable Daily Intake (µg/Kg_bw_/day) for plasticizers and bisphenols from provola samples.

	EDI		HQ		TDI
	CTR	BIO	CTR	BIO	
**Plasticizers**					
DMA	4.0 × 10^−03^ ± 7.5 × 10^−04^	3.9 × 10^−03^ ± 8.3 × 10^−04^	-	-	n.a.
DMP	3.3 × 10^−03^ ± 2.9 × 10^−04^	3.3 × 10^−03^ ± 3.2 × 10^−04^	-	-	n.a.
DEP	4.6 × 10^−03^ ± 6.6 × 10^−04^	4.7 × 10^−03^ ± 6.2 × 10^−04^	9.2 × 10^−07^ ± 1.3 × 10^−07^	9.3 × 10^−07^ ± 1.2 × 10^−07^	5000
DPrP	2.3 × 10^−03^ ± 2.0 × 10^−04^	2.2 × 10^−03^ ± 1.8 × 10^−04^	-	-	n.a.
DiBP	1.2 × 10^−02^ ± 1.5 × 10^−03^	1.1 × 10^−02^ ± 1.4 × 10^−03^	-	-	n.a.
DBP	5.0 × 10^−03^ ± 9.9 × 10^−04^	5.0 × 10^−03^ ± 9.5 × 10^−04^	5.0 × 10^−04^ ± 9.9 × 10^−05^	5.0 × 10^−04^ ± 9.5 × 10^−05^	10
DEHA	5.7 × 10^−02^ ± 1.9 × 10^−02^	5.7 × 10^−02^ ± 1.9 × 10^−02^	1.9 × 10^−04^ ± 6.4 × 10^−05^	1.9 × 10^−04^ ± 6.4 × 10^−05^	300
DEHP	1.4 × 10^−02^ ± 2.1 × 10^−03^	1.4 × 10^−02^ ± 1.7 × 10^−03^	2.8 × 10^−04^ ± 4.1 × 10^−05^	2.9 × 10^−04^ ± 3.4 × 10^−05^	50
DEHT	5.1 × 10^−02^ ± 2.3 × 10^−02^	5.0 × 10^−02^ ± 2.1 × 10^−02^	-	-	n.a.
∑DBP and DEHP	1.9 × 10^−02^ ± 1.8 × 10^−03^	1.9 × 10^−02^ ± 1.5 × 10^−03^	3.8 × 10^−04^ ± 3.7 × 10^−05^	3.8 × 10^−04^ ± 3.0 × 10^−05^	50
**Bisphenols**					
BPAF	1.9 × 10^−03^ ± 2.7 × 10^−04^	1.9 × 10^−03^ ± 1.0 × 10^−04^	-	-	n.a.
BPS	1.7 × 10^−03^ ± 2.4 × 10^−04^	1.6 × 10^−03^ ± 1.4 × 10^−04^	-	-	n.a.

n.a., not available.

## Data Availability

Data is contained within the article or supplementary material.

## Supplementary Materials

The following supporting information can be downloaded at: https://www.mdpi.com/article/10.3390/foods11233817/s1, Table S1. The name, chemical class and sales companies of the 140 persistent organic pollutants under analysis. Table S2. Ingredients and nutritional characteristics of the concentrates. Table S3. GC-MS/MS acquisition parameters for the 140 persistent organic pollutants under analysis. Table S4. Linearity, LOD, LOQ, for the 140 persistent organic pollutants under analysis. Table S5. List of the investigated plasticizers. tr: Retention Time; T: target ion; Q1 e Q2:qualifying ions; linearity, LOD, LOQ. Table S6. List of the investigated bisphenols with Retention Time and Monitored ions (*m/z*), linearity, LOD, LOQ. Table S7. Estimated Daily Intake (µg/Kg_bw_/day), Hazard Index and Tolerable Daily Intake (µg/Kg_bw_/day) for plasticizers and bisphenols from Provola samples categorized by diet and period.
