# Supplementary material for: Evaluation of Chemical Contaminants in Conventional and Unconventional Ragusana Provola Cheese
Source: Foods. 2022 Nov 26;11(23):3817. doi: 10.3390/foods11233817 (PMC9740842; doi:10.3390/foods11233817)
Supplement: Supplementary file 1 [file foods-11-03817-s001.zip › foods-2007794-supplementary - Revised.pdf]

foods MDPI

Supplementary materials

Table S1. The name, chemical class and sales companies of the 140 persistent organic pollutants under analysis.

Compound	Chemical class	Sales companies
(±)-Indoxacarb	Carbamates (Cs)	Dr. Ehrenstorfer (Augsburg, Germany)
Bendiocarb	Carbamates (Cs)	Dr. Ehrenstorfer (Augsburg, Germany)
Carbaryl	Carbamates (Cs)	Dr. Ehrenstorfer (Augsburg, Germany)
Carbofuran	Carbamates (Cs)	Dr. Ehrenstorfer (Augsburg, Germany)
Carbophenothion	Carbamates (Cs)	Dr. Ehrenstorfer (Augsburg, Germany)
Diethofencarb	Carbamates (Cs)	Dr. Ehrenstorfer (Augsburg, Germany)
Ethiofencarb	Carbamates (Cs)	Dr. Ehrenstorfer (Augsburg, Germany)
Furathiocarb	Carbamates (Cs)	Dr. Ehrenstorfer (Augsburg, Germany)
Phenoxycarb	Carbamates (Cs)	Dr. Ehrenstorfer (Augsburg, Germany)
Pirimicarb	Carbamates (Cs)	Dr. Ehrenstorfer (Augsburg, Germany)
Mecarbam	Carbamates (Cs)/Acaricides (As)	Dr. Ehrenstorfer (Augsburg, Germany)
Azoxystrobin	Fungicides (Fs)	Dr. Ehrenstorfer (Augsburg, Germany)
Boscalid	Fungicides (Fs)	Dr. Ehrenstorfer (Augsburg, Germany)
Bupirimate	Fungicides (Fs)	Dr. Ehrenstorfer (Augsburg, Germany)
Captafol	Fungicides (Fs)	Dr. Ehrenstorfer (Augsburg, Germany)
Captan	Fungicides (Fs)	Dr. Ehrenstorfer (Augsburg, Germany)
Cyproconazole isomer II	Fungicides (Fs)	Dr. Ehrenstorfer (Augsburg, Germany)
Diclobutrazol	Fungicides (Fs)	Dr. Ehrenstorfer (Augsburg, Germany)
Fenarimol	Fungicides (Fs)	Dr. Ehrenstorfer (Augsburg, Germany)
Fenhexamid	Fungicides (Fs)	Dr. Ehrenstorfer (Augsburg, Germany)
Fluodioxonil	Fungicides (Fs)	Dr. Ehrenstorfer (Augsburg, Germany)
Flusilazole	Fungicides (Fs)	Dr. Ehrenstorfer (Augsburg, Germany)
Imazalil	Fungicides (Fs)	Dr. Ehrenstorfer (Augsburg, Germany)
Kresoxim mehyl	Fungicides (Fs)	Dr. Ehrenstorfer (Augsburg, Germany)
Metalaxyl-M	Fungicides (Fs)	Dr. Ehrenstorfer (Augsburg, Germany)
Mepronil	Fungicides (Fs)	Dr. Ehrenstorfer (Augsburg, Germany)
Penconazole	Fungicides (Fs)	Dr. Ehrenstorfer (Augsburg, Germany)
Prochloraz	Fungicides (Fs)	Dr. Ehrenstorfer (Augsburg, Germany)
Procymidone	Fungicides (Fs)	Dr. Ehrenstorfer (Augsburg, Germany)
Pyrimethanil	Fungicides (Fs)	Dr. Ehrenstorfer (Augsburg, Germany)
Quintozen	Fungicides (Fs)	Dr. Ehrenstorfer (Augsburg, Germany)
Tebuconazole	Fungicides (Fs)	Dr. Ehrenstorfer (Augsburg, Germany)
Tolchlophos methyl	Fungicides (Fs)	Dr. Ehrenstorfer (Augsburg, Germany)
Triadimefon	Fungicides (Fs)	Dr. Ehrenstorfer (Augsburg, Germany)
Trifloxystrobin	Fungicides (Fs)	Dr. Ehrenstorfer (Augsburg, Germany)
Vinclozolin	Fungicides (Fs)	Dr. Ehrenstorfer (Augsburg, Germany)
Amandryn	Herbicides (Hs)	Dr. Ehrenstorfer (Augsburg, Germany)
Atrazine	Herbicides (Hs)	Dr. Ehrenstorfer (Augsburg, Germany)
Diflufenican	Herbicides (Hs)	Dr. Ehrenstorfer (Augsburg, Germany)
Linuron	Herbicides (Hs)	Dr. Ehrenstorfer (Augsburg, Germany)
Methabenzthiazuron	Herbicides (Hs)	Dr. Ehrenstorfer (Augsburg, Germany)
Oxyfluorfen	Herbicides (Hs)	Dr. Ehrenstorfer (Augsburg, Germany)
Propazine	Herbicides (Hs)	Dr. Ehrenstorfer (Augsburg, Germany)
Propyzamide	Herbicides (Hs)	Dr. Ehrenstorfer (Augsburg, Germany)
Simazine	Herbicides (Hs)	Dr. Ehrenstorfer (Augsburg, Germany)
Terbuthilazine	Herbicides (Hs)	Dr. Ehrenstorfer (Augsburg, Germany)
Trifluralin	Herbicides (Hs)	Dr. Ehrenstorfer (Augsburg, Germany)
Buprofezin	Insect growth regulators (IGRs)	Dr. Ehrenstorfer (Augsburg, Germany)
Cyromazine	Insect growth regulators (IGRs)	Dr. Ehrenstorfer (Augsburg, Germany)
Pyriproxyfen	Insect growth regulators (IGRs)	Dr. Ehrenstorfer (Augsburg, Germany)
2,4′-DDD	Organochlorine pesticides (OCPs)	Fluka Analytical (Milan, Italy)
2,4′-DDE	Organochlorine pesticides (OCPs)	Fluka Analytical (Milan, Italy)
2,4′-DDT	Organochlorine pesticides (OCPs)	Fluka Analytical (Milan, Italy)
4,4′-DDD	Organochlorine pesticides (OCPs)	Fluka Analytical (Milan, Italy)
4,4′-DDE	Organochlorine pesticides (OCPs)	Fluka Analytical (Milan, Italy)
4,4′-DDT	Organochlorine pesticides (OCPs)	Fluka Analytical (Milan, Italy)

Compound	Chemical class	Sales companies
Alachlor	Organochlorine pesticides (OCPs)	Fluka Analytical (Milan, Italy)
Aldrin	Organochlorine pesticides (OCPs)	Fluka Analytical (Milan, Italy)
cis-Chlordane	Organochlorine pesticides (OCPs)	Fluka Analytical (Milan, Italy)
Dicofol	Organochlorine pesticides (OCPs)	Fluka Analytical (Milan, Italy)
Dieldrin	Organochlorine pesticides (OCPs)	Fluka Analytical (Milan, Italy)
Endosulfan sulfate	Organochlorine pesticides (OCPs)	Fluka Analytical (Milan, Italy)
Endosulfan α	Organochlorine pesticides (OCPs)	Fluka Analytical (Milan, Italy)
Endosulfan β	Organochlorine pesticides (OCPs)	Fluka Analytical (Milan, Italy)
Endrin	Organochlorine pesticides (OCPs)	Fluka Analytical (Milan, Italy)
Methoxychlor	Organochlorine pesticides (OCPs)	Dr. Ehrenstorfer (Augsburg, Germany)
trans-Chlordane	Organochlorine pesticides (OCPs)	Fluka Analytical (Milan, Italy)
α-HCH	Organochlorine pesticides (OCPs)	Fluka Analytical (Milan, Italy)
β-HCH	Organochlorine pesticides (OCPs)	Fluka Analytical (Milan, Italy)
γ-HCH	Organochlorine pesticides (OCPs)	Fluka Analytical (Milan, Italy)
Acephate	Organophosphorous pesticides (OPPs)	Dr. Ehrenstorfer (Augsburg, Germany)
Azinphos ethyl	Organophosphorous pesticides (OPPs)	Dr. Ehrenstorfer (Augsburg, Germany)
Bromophos methyl	Organophosphorous pesticides (OPPs)	Dr. Ehrenstorfer (Augsburg, Germany)
Chlorpyriphos	Organophosphorous pesticides (OPPs)	Dr. Ehrenstorfer (Augsburg, Germany)
Chlorpyriphos methyl	Organophosphorous pesticides (OPPs)	Dr. Ehrenstorfer (Augsburg, Germany)
cis-Chlorfenvinphos	Organophosphorous pesticides (OPPs)	Dr. Ehrenstorfer (Augsburg, Germany)
Coumaphos	Organophosphorous pesticides (OPPs)	Dr. Ehrenstorfer (Augsburg, Germany)
Diazinon	Organophosphorous pesticides (OPPs)	Dr. Ehrenstorfer (Augsburg, Germany)
Dimethoate	Organophosphorous pesticides (OPPs)	Dr. Ehrenstorfer (Augsburg, Germany)
Ethion	Organophosphorous pesticides (OPPs)	Dr. Ehrenstorfer (Augsburg, Germany)
Fenamiphos	Organophosphorous pesticides (OPPs)	Dr. Ehrenstorfer (Augsburg, Germany)
Fenchlorphos	Organophosphorous pesticides (OPPs)	Dr. Ehrenstorfer (Augsburg, Germany)
Fenitrothion	Organophosphorous pesticides (OPPs)	Dr. Ehrenstorfer (Augsburg, Germany)
Fenthion	Organophosphorous pesticides (OPPs)	Dr. Ehrenstorfer (Augsburg, Germany)
Fenthion Sulfone	Organophosphorous pesticides (OPPs)	Dr. Ehrenstorfer (Augsburg, Germany)
Fenthion Sulfoxide	Organophosphorous pesticides (OPPs)	Dr. Ehrenstorfer (Augsburg, Germany)
Malathion	Organophosphorous pesticides (OPPs)	Dr. Ehrenstorfer (Augsburg, Germany)
Methidathion	Organophosphorous pesticides (OPPs)	Dr. Ehrenstorfer (Augsburg, Germany)
Omethoate	Organophosphorous pesticides (OPPs)	Dr. Ehrenstorfer (Augsburg, Germany)
Parathion methyl	Organophosphorous pesticides (OPPs)	Dr. Ehrenstorfer (Augsburg, Germany)
Phenthoate	Organophosphorous pesticides (OPPs)	Dr. Ehrenstorfer (Augsburg, Germany)
Phosalone	Organophosphorous pesticides (OPPs)	Dr. Ehrenstorfer (Augsburg, Germany)
Phosmet	Organophosphorous pesticides (OPPs)	Dr. Ehrenstorfer (Augsburg, Germany)
Phoxim	Organophosphorous pesticides (OPPs)	Dr. Ehrenstorfer (Augsburg, Germany)
Quinalphos	Organophosphorous pesticides (OPPs)	Dr. Ehrenstorfer (Augsburg, Germany)
trans-Chlorfenvinphos	Organophosphorous pesticides (OPPs)	Dr. Ehrenstorfer (Augsburg, Germany)
Triphenyl phosphate	Organophosphorous pesticides (OPPs)	Aldrich Chemical (Chicago, Il, USA)
Carbophenothion	Organophosphorous pesticides (OPPs)/Acaricides (As)	Dr. Ehrenstorfer (Augsburg, Germany)
Pirimiphos-methyl	Organophosphorous pesticides (OPPs)/Acaricides (As)	Dr. Ehrenstorfer (Augsburg, Germany)
PCB28	Polychlorobiphenyls (PCBs)	Aldrich Chemical (Chicago, Il, USA)
PCB52	Polychlorobiphenyls (PCBs)	Aldrich Chemical (Chicago, Il, USA)
PCB77	Polychlorobiphenyls (PCBs)	Aldrich Chemical (Chicago, Il, USA)
PCB81	Polychlorobiphenyls (PCBs)	Aldrich Chemical (Chicago, Il, USA)
PCB101	Polychlorobiphenyls (PCBs)	Aldrich Chemical (Chicago, Il, USA)
PCB105	Polychlorobiphenyls (PCBs)	Aldrich Chemical (Chicago, Il, USA)
PCB114	Polychlorobiphenyls (PCBs)	Aldrich Chemical (Chicago, Il, USA)
PCB118	Polychlorobiphenyls (PCBs)	Aldrich Chemical (Chicago, Il, USA)
PCB123	Polychlorobiphenyls (PCBs)	Aldrich Chemical (Chicago, Il, USA)
PCB126	Polychlorobiphenyls (PCBs)	Aldrich Chemical (Chicago, Il, USA)
PCB138	Polychlorobiphenyls (PCBs)	Aldrich Chemical (Chicago, Il, USA)
PCB153	Polychlorobiphenyls (PCBs)	Aldrich Chemical (Chicago, Il, USA)
PCB156	Polychlorobiphenyls (PCBs)	Aldrich Chemical (Chicago, Il, USA)
PCB157	Polychlorobiphenyls (PCBs)	Aldrich Chemical (Chicago, Il, USA)
PCB167	Polychlorobiphenyls (PCBs)	Aldrich Chemical (Chicago, Il, USA)
PCB169	Polychlorobiphenyls (PCBs)	Aldrich Chemical (Chicago, Il, USA)
PCB180	Polychlorobiphenyls (PCBs)	Aldrich Chemical (Chicago, Il, USA)
PCB189	Polychlorobiphenyls (PCBs)	Aldrich Chemical (Chicago, Il, USA)

Compound	Chemical class	Sales companies
Acenaphthylene	Polycyclic aromatic hydrocarbons (PAHs)	Aldrich Chemical (Chicago, Il, USA)
Anthracene	Polycyclic aromatic hydrocarbons (PAHs)	Aldrich Chemical (Chicago, Il, USA)
Benzo[a]anthracene	Polycyclic aromatic hydrocarbons (PAHs)	Aldrich Chemical (Chicago, Il, USA)
Benzo[a]pyrene	Polycyclic aromatic hydrocarbons (PAHs)	Aldrich Chemical (Chicago, Il, USA)
Benzo[b]fluoranthene	Polycyclic aromatic hydrocarbons (PAHs)	Aldrich Chemical (Chicago, Il, USA)
Benzo[g,h,i]perylene	Polycyclic aromatic hydrocarbons (PAHs)	Aldrich Chemical (Chicago, Il, USA)
Benzo[k]fluoranthene	Polycyclic aromatic hydrocarbons (PAHs)	Aldrich Chemical (Chicago, Il, USA)
Chrysene	Polycyclic aromatic hydrocarbons (PAHs)	Aldrich Chemical (Chicago, Il, USA)
Dibenzo[a,h]anthracene	Polycyclic aromatic hydrocarbons (PAHs)	Aldrich Chemical (Chicago, Il, USA)
Fluorene	Polycyclic aromatic hydrocarbons (PAHs)	Aldrich Chemical (Chicago, Il, USA)
Indeno[1,2,3-cd]pyrene	Polycyclic aromatic hydrocarbons (PAHs)	Aldrich Chemical (Chicago, Il, USA)
Phenanthrene	Polycyclic aromatic hydrocarbons (PAHs)	Aldrich Chemical (Chicago, Il, USA)
Pyrene	Polycyclic aromatic hydrocarbons (PAHs)	Aldrich Chemical (Chicago, Il, USA)
cis-Fluvalinate	Pyrethroid insecticides (PYRs)	Dr. Ehrenstorfer (Augsburg, Germany)
cis-Permandhrin	Pyrethroid insecticides (PYRs)	Dr. Ehrenstorfer (Augsburg, Germany)
Cypermethrin isomer I	Pyrethroid insecticides (PYRs)	Dr. Ehrenstorfer (Augsburg, Germany)
Cypermethrin isomer II	Pyrethroid insecticides (PYRs)	Dr. Ehrenstorfer (Augsburg, Germany)
Cypermethrin isomer III	Pyrethroid insecticides (PYRs)	Dr. Ehrenstorfer (Augsburg, Germany)
Deltamethrin	Pyrethroid insecticides (PYRs)	Dr. Ehrenstorfer (Augsburg, Germany)
trans-Fluvalinate	Pyrethroid insecticides (PYRs)	Dr. Ehrenstorfer (Augsburg, Germany)
trans-Permethrin	Pyrethroid insecticides (PYRs)	Dr. Ehrenstorfer (Augsburg, Germany)
Λ-Cyhalothrin	Pyrethroid insecticides (PYRs)	Dr. Ehrenstorfer (Augsburg, Germany)
Piperonyl butoxide	Synergists (SYNs)	Dr. Ehrenstorfer (Augsburg, Germany)

Table S2. Ingredients and nutritional characteristics of the concentrates.

	CTR	BIO
Ingredient, kg of DM		
Corn meal	390	390
Soybean meal (48% CP)	190	180
Barley meal	150	115
Wheat middling	120	115
Sunflower meal	60.0	50.0
Dried Olive Cake	-	80.0
Beet pulp	60.0	40.0
Calcium carbonate	7.0	7.0
Na bicarbonate	5.0	5.0
Na chloride	5.0	5.0
P dicalcium	4.0	4.0
Mg oxide	3.0	3.0
Na propionate	1.0	1.0
Vitamin premix*	5.0	5.0
Chemical composition, g/kg of DM		
Moisture	106	113
Starch	396.5	403.8
Crude protein	168	170
Crude oil and fats	40.7	41.2
Neutral detergent fibre	217.6	239.4
Acid detergent fibre	89.2	131.8
Acid detergent lignin	21.3	48.3
Ash	76.8	76.3

*Providing per kg of diet: 32,000U vitamin A,3,200U Vitamin D_3,120 mg Vitamin E, 8mg Vitamin B_1, 1.6mg Vitamin B_2, 0,016 Vitamin B_{12}, 400mg Niacin, 4mg Pantothenic acid, 400mg choline chloride.

Table S3. GC-MS/MS acquisition parameters for the 140 persistent organic pollutants under analysis.

Chemical class	Compound	Reaction 1	CE 1	Reaction 2	CE 2
Carbamates (Cs)					
	(±)-Indoxacarb	218 → 203	10	218 → 134	20
	Bendiocarb	166 → 151	10	166 → 109	20
	Carbaryl	144 → 115	20	115 → 89	20
	Carbofuran	164 → 149	15	164 → 103	20
	Carbophenothion	157 → 121	20	157 → 77	20
	Diethofencarb	267 → 225	10	267 → 168	20
	Ethiofencarb	168 → 107	10	168 → 77	25
	Furathiocarb	194 → 105	20	194 → 165	15
	Phenoxycarb	116 → 88	15	186 → 109	15
	Pirimicarb	238 → 166	10	206 → 166	15
Carbamates (Cs)/Acaricides (As)					
	Mecarbam	296 → 196	10	296 → 168	10
Fungicides (Fs)					
	Azoxystrobin	344 → 329	20	344 → 183	20
	Boscalid	342 → 140	10	342 → 112	25
	Bupirimate	208 → 165	15	108 → 140	15
	Captafol	151 → 79	20	151 → 122	10
	Captan	107 → 79	10	107 → 77	20
	Cyproconazole isomer II	222 → 125	20	224 → 127	20
	Diclobutrazol	270 → 159	10	270 → 137	25
	Fenarimol	251 → 139	20	251 → 111	25
	Fenhexamid	177 → 78	20	177 → 113	20
	Fluodioxonil	248 → 127	20	248 → 154	25
	Flusilazole	233 → 165	20	233 → 152	20
	Imazalil	215 → 173	15	215 → 145	25
	Kresoxim methyl	206 → 131	10	206 → 116	10
	Metalaxyl-M	160 → 130	20	160 → 144	20
	Mepronil	269 → 119	10	210 → 181	20
	Penconazole	248 → 157	20	248 → 192	20
	Prochloraz	180 → 138	15	180 → 69	20
	Procymidone	283 → 96	10	285 → 96	15
	Pyrimethanil	198 → 118	30	199 → 198	25
	Quintozen	237 → 143	20	237 → 119	20
	Tebuconazole	250 → 125	15	125 → 89	25
	Tolchlophos methyl	265 → 250	20	265 → 93	24
	Triadimefon	208 → 181	10	208 → 127	15
	Trifloxystrobin	190 → 130	15	190 → 102	25
	Vinclozolin	212 → 177	15	212 → 145	20
Herbicides (Hs)					
	Amandryn	227 → 170	10	227 → 185	10
	Atrazine	200 → 122	15	215 → 200	10
	Diflufenican	266 → 183	25	246 → 218	25
	Linuron	160 → 133	15	160 → 125	15
	Methabenzthiazuron	164 → 136	15	127 → 109	20
	Oxyfluorfen	300 → 223	20	252 → 170	25
	Propazine	214 → 172	15	214 → 94	20
	Propyzamide	173 → 145	15	173 → 109	25
	Simazine	201 → 173	7	201 → 186	8
	Terbuthilazine	214 → 104	15	214 → 132	10
	Trifluralin	264 → 160	15	264 → 206	10
Insect growth regulators (IGRs)					
	Buprofezin	175 → 132	15	175 → 117	20
	Cyromazine	151 → 109	15	165 → 123	20
	Pyriproxyfen	136 → 78	20	136 → 96	20
Organochlorine pesticides (OCPs)					
	2,4′-DDD	235 → 165	20	237 → 165	20
	2,4′-DDE	246 → 176	20	318 → 248	20
	2,4′-DDT	235 → 165	20	237 → 165	20
	4,4′-DDD	235 → 165	20	237 → 165	20

Chemical class	Compound	Reaction 1	CE 1	Reaction 2	CE 2
	4,4′-DDE	246 → 176	30	318 → 248	30
	4,4′-DDT	235 → 165	20	237 → 165	20
	Alachlor	188 → 160	15	161 → 146	15
	Aldrin	263 → 193	20	293 → 258	20
	cis-Chlordane	373 → 266	20	373 → 264	20
	Dicofol	250 → 139	20	250 → 215	10
	Dieldrin	263 → 193	20	263 → 228	20
	Endosulfan sulfate	272 → 237	15	274 → 239	15
	Endosulfan α	241 → 206	25	241 → 170	25
	Endosulfan β	195 → 160	10	195 → 125	20
	Endrin	263 → 193	20	281 → 245	15
	Methoxychlor	227 → 169	20	227 → 141	25
	trans-Chlordane	373 → 266	20	373 → 264	20
	α-HCH	181 → 145	10	219 → 183	10
	β-HCH	181 → 145	15	219 → 183	10
	γ-HCH	181 → 145	15	219 → 183	10
Organophosphorous pesticides (OPPs)					
	Acephate	136 → 94	10	136 → 119	8
	Andhion	231 → 175	15	231 → 129	20
	Azinphos ethyl	160 → 132	5	160 → 77	10
	Chlorpyriphos	197 → 169	15	197 → 169	15
	Chlorpyriphos methyl	286 → 93	25	286 → 271	20
	cis-Chlorfenvinphos	267 → 159	20	269 → 161	20
	Coumaphos	226 → 163	20	226 → 135	25
	Diazinon	137 → 84	15	179 → 137	20
	Dimethoate	125 → 79	20	125 → 79	8
	Fenamiphos	303 → 154	15	303 → 195	10
	Fenchlorphos	285 → 270	20	285 → 240	20
	Fenitrothion	125 → 79	15	277 → 125	18
	Fenthion	278 → 109	20	278 → 125	22
	Fenthion Sulfone	310 → 105	20	310 → 109	30
	Fenthion Sulfoxide	278 → 109	15	278 → 169	25
	Malathion	173 → 99	15	173 → 117	15
	Methidathion	145 → 85	10	145 → 58	20
	Omethoate	156 → 110	10	156 → 79	30
	Parathion methyl	263 → 109	15	263 → 246	6
	Phenthoate	274 → 125	15	274 → 121	15
	Phosalone	182 → 111	20	182 → 75	30
	Phosmet	160 → 77	25	160 → 133	15
	Phoxim	109 → 81	15	109 → 91	15
	Quinalphos	146 → 118	15	146 → 91	30
	trans-Chlorfenvinphos	267 → 159	20	269 → 161	20
	Triphenyl phosphate	325 → 169	20	325 → 77	25
Organophosphorous pesticides (OPPs)/Acaricides (As)					
	Carbophenothion	157 → 121	20	157 → 77	20
	Pirimiphos-methyl	290 → 125	15	290 → 151	15
Polychlorobiphenyls (PCBs)					
	PCB28	256 → 186	15	258 → 186	15
	PCB52	290 → 220	15	292 → 222	15
	PCB77	290 → 220	20	292 → 222	20
	PCB81	290 → 220	20	292 → 222	20
	PCB101	324 → 254	20	326 → 256	20
	PCB105	324 → 254	20	326 → 256	20
	PCB114	324 → 254	20	326 → 256	20
	PCB118	324 → 254	20	326 → 256	20
	PCB123	324 → 254	20	326 → 256	20
	PCB126	324 → 254	20	326 → 256	20
	PCB138	360 → 290	25	362 → 292	25
	PCB153	360 → 290	25	362 → 292	25
	PCB156	360 → 290	30	362 → 292	30
	PCB157	360 → 290	30	362 → 292	30
	PCB167	360 → 290	30	362 → 292	30

Chemical class	Compound	Reaction 1	CE 1	Reaction 2	CE 2
	PCB169	360 → 290	30	362 → 292	30
	PCB180	394 → 324	20	396 → 326	20
	PCB189	394 → 324	25	396 → 326	25
Polycyclic aromatic hydrocarbons (PAHs)					
	Acenaphthylene	152 → 126	30	152 → 102	30
	Anthracene	178 → 152	25	176 → 150	25
	Benzo[a]anthracene	228 → 226	30	228 → 202	20
	Benzo[a]pyrene	252 → 250	35	252 → 226	20
	Benzo[b]fluoranthene	252 → 250	35	126 → 113	10
	Benzo[g,h,i]perylene	276 → 274	45	276 → 272	50
	Benzo[k]fluoranthene	252 → 250	35	126 → 113	10
	Chrysene	228 → 226	30	228 → 202	20
	Dibenzo[a,h]anthracene	278 → 276	30	278 → 252	20
	Fluorene	166 → 165	15	165 → 164	20
	Indeno[1,2,3-cd]pyrene	276 → 274	30	137 → 136	15
	Phenanthrene	178 → 152	25	176 → 150	25
	Pyrene	202 → 200	20	202 → 152	30
Pyrethroid insecticides (PYRs)					
	cis-Fluvalinate	250 → 55	15	252 → 55	20
	cis-Permethrin	183 → 153	15	183 → 168	15
	Cypermethrin isomer I	181 → 152	20	163 → 91	15
	Cypermethrin isomer II	181 → 152	20	163 → 91	15
	Cypermethrin isomer III	181 → 152	20	163 → 91	15
	Deltamethrin	181 → 152	20	253 → 93	15
	trans-Fluvalinate	250 → 55	15	252 → 55	20
	trans-Permethrin	183 → 153	20	183 → 168	20
	Λ-Cyhalothrin	181 → 152	25	197 → 141	10
Synergists (SYNs)					
	Piperonyl butoxide	176 → 131	15	176 → 103	20

Table S4. Linearity, LOD, LOQ, for the 140 persistent organic pollutants under analysis.

Compound	R^2	LOD (ng/g)	LOQ (ng/g)
(±)-Indoxacarb	0.995	0.14	0.51
2,4′-DDD	1.000	0.02	0.04
2,4′-DDE	0.996	0.08	0.21
2,4′-DDT	0.996	0.05	0.11
4,4′-DDD	0.998	0.05	0.15
4,4′-DDE	0.994	0.04	0.12
4,4′-DDT	0.997	0.08	0.23
Acenaphthylene	0.980	0.10	0.29
Acephate	0.990	0.15	0.56
Alachlor	0.998	0.04	0.14
Aldrin	0.992	1.14	4.21
Ametryn	0.989	0.06	0.2
Anthracene	0.987	0.13	0.42
Atrazine	0.994	0.93	3.61
Azinphos ethyl	1.000	0.03	0.09
Azoxystrobin	0.986	1.32	4.37
Bendiocarb	0.998	0.06	0.17
Benzo[a]anthracene	0.955	0.12	0.38
Benzo[a]pyrene	0.985	0.32	1.05
Benzo[b]fluoranthene	0.922	2.54	8.38
Benzo[ghi]perylene	0.972	0.42	1.31
Benzo[k]fluoranthene	0.980	2.55	8.36
Boscalid	0.989	0.26	0.86
Bupirimate	0.990	1.38	4.23
Buprofezin	0.995	2.32	8.22
Captafol	0.992	0.07	0.23
Captan	0.992	0.41	1.22
Carbaryl	0.982	0.82	2.92
Carbofuran	0.996	0.13	0.48
Carbophenthion	0.986	0.25	0.82
Chlorpyriphos	1.000	0.12	0.36
Chlorpyriphos methyl	0.999	0.15	0.47
Chrysene	0.972	0.35	1.17
cis-Chlordane	0.994	0.11	0.34
cis-Chlorfenvinphos	0.992	0.31	0.99
cis-Fluvalinate	0.998	2.45	8.24
cis-Permethrin	0.994	2.49	8.18
Coumaphos	0.996	0.12	0.4
Cypermethrin isomer I	0.999	5.09	14.5
Cypermethrin isomer II	0.984	5.51	19.3
Cypermethrin isomer III	0.992	4.85	15.8
Cyproconazole isomer II	0.978	0.32	1.29
Cyromazine	0.952	0.54	1.62
Deltamethrin	0.999	0.11	0.33
Diethofencarb	0.998	0.05	0.17
Diazinon	1.000	0.12	0.38
Dibenz[a,h]anthracene	0.961	5.11	17.08
Diclobutrazol	0.989	0.18	0.65
Dicofol	0.999	0.06	0.18
Dieldrin	0.990	0.16	0.54
Diflufenican	0.996	0.09	0.31
Dimethoate	0.962	0.52	1.62
Endosulfan sulfate	0.996	0.21	0.65
Endosulfan α	0.998	0.09	0.29
Endosulfan β	0.996	0.16	0.55
Endrin	0.998	2.33	8.23
Ethiofencarb	0.985	0.26	0.95
Ethion	0.986	0.82	2.85
Fenamiphos	0.992	0.19	0.62
Fenarimol	0.998	0.12	0.36
Fenchlorphos	0.996	0.12	0.53

Compound	R^2	LOD (ng/g)	LOQ (ng/g)
Fenhexamid	0.998	0.09	0.31
Fenitrothion	0.998	0.15	0.46
Fenthion	1.000	0.07	0.23
Fenthion Sulfone	0.972	0.09	0.29
Fenthion Sulfoxide	0.978	0.12	0.38
Fludioxonil	0.980	0.14	0.42
Fluorene	0.985	0.04	0.14
Flusilazole	0.986	0.28	0.94
Furathiocarb	0.998	0.12	0.42
Imazalil	0.987	0.35	1.13
Indenopyrene	0.969	0.36	1.15
Kresoxim methyl	0.999	0.29	0.91
Linuron	0.996	1.68	6.04
Malathion	0.984	0.76	2.64
Metalaxyl-M	0.999	0.08	0.27
Methabenzthiazuron	0.984	0.29	0.92
Methidathion	0.992	0.09	0.28
Methoxychlor	0.999	0.11	0.35
Mecarbam	0.986	0.09	0.29
Mepronil	0.998	0.07	0.21
Omethoate	0.990	0.07	0.23
Oxyfluorfen	0.998	0.19	0.58
Parathion methyl	0.970	0.69	2.55
PCB28	0.995	0.04	0.13
PCB52	0.992	0.09	0.28
PCB77	1.000	0.07	0.20
PCB81	0.994	0.12	0.35
PCB101	0.999	0.06	0.16
PCB105	1.000	0.11	0.30
PCB114	0.997	0.04	0.10
PCB118	1.000	0.04	0.10
PCB123	0.998	0.10	0.29
PCB126	0.999	0.07	0.18
PCB138	1.000	0.11	0.29
PCB153	0.997	0.13	0.30
PCB156	1.000	0.11	0.37
PCB157	0.998	0.14	0.46
PCB167	0.999	0.12	0.38
PCB169	0.999	0.09	0.30
PCB180	1.000	0.08	0.22
PCB189	1.000	0.08	0.24
Penconazole	0.996	0.06	0.22
Phenanthrene	0.989	0.12	0.35
Phenoxycarb	0.996	0.14	0.49
Phenthoate	0.999	0.09	0.32
Phosalone	0.998	0.08	0.24
Phosmet	0.982	0.34	1.15
Phoxim	0.982	0.13	0.46
Piperonyl butoxide	1.000	0.24	0.92
Pirimicarb	1.000	0.06	0.19
Pirimiphos-methyl	0.978	0.74	2.75
Prochloraz	0.996	0.33	1.13
Procymidone	0.996	0.14	0.34
Propazine	0.992	0.15	0.52
Propyzamide	0.998	0.04	0.14
Pyrene	0.990	0.11	0.24
Pyrimethanil	0.990	0.13	0.38
Pyriproxyfen	0.994	0.12	0.41
Quinalphos	0.996	0.09	0.28
Quintozen	0.990	0.28	0.86
Simazine	1.000	0.1	0.31
Tebuconazole	0.994	0.17	0.48
Terbuthilazine	0.990	0.1	0.34

Compound	R^2	LOD (ng/g)	LOQ (ng/g)
Tolchlophos methyl	1.000	0.11	0.32
trans-Chlordane	0.996	0.12	0.42
trans-Chlorfenvinphos	0.997	0.09	0.38
trans-Fluvalinate	0.999	2.42	8.02
trans-Permandhrin	0.996	2.38	8.21
Triadimefon	0.990	0.39	1.18
Trifloxystrobin	0.994	0.52	1.76
Trifloxystrobin	0.992	0.08	0.26
Trifluralin	1.000	0.1	0.3
Triphenyl phosphate	0.999	0.05	0.14
Vinclozolin	0.999	0.1	0.33
α-HCH	0.994	0.1	0.32
β-HCH	0.986	0.09	0.29
γ-HCH	0.988	0.11	0.37
Λ-Cyhalothrin	1.000	0.06	0.18

Table S5. List of the investigated plasticizers. tr: Retention Time; T: target ion; Q1 e Q2:qualifying ions; linearity, LOD, LOQ.

Compound	Abbreviation	t_r(min)	T, Q1, Q2 (m/z)	r^2	LOD (mg/Kg)	LOQ
dimethyl phthalate	DMP	13.7	163, 92,164	0.9954	0.007	0.023
diethyl phthalate	DEP	15.8	149, 177, 176	0.9923	0.005	0.017
dipropyl phthalate	DPrP	18.8	149, 150, 209	0.9939	0.007	0.020
dibutyl phthalate	DBP	23.3	149, 150, 223	0.9948	0.007	0.023
diisobutyl phthalate	DiBP	20.9	149, 150, 223	0.9921	0.007	0.023
butyl benzyl phthalate	BBP	30.2	149, 91, 206	0.9883	0.037	0.121
diphenyl phthalate	DPhP	34.4	225, 226, 104	0.9945	0.015	0.051
dicyclohexyl phthalate	DcHexP	33.8	149, 167, 150	0.9985	0.027	0.087
diheptyl phthalate	DHepP	31.9	149, 99, 265	0.9954	0.177	0.553
di(2-ethylhexyl) phthalate	DEHP	34.1	149, 167, 279	0.9988	0.007	0.020
dimethyl adipate	DMA	10.1	114, 101, 111	0.9965	0.010	0.030
diethyl adipate	DEA	12.5	111, 157, 128	0.9943	0.013	0.037
benzyl benzoate	BB	19.0	105, 91, 212	0.9899	0.012	0.033
dibutyl adipate	DBA	18.6	129, 185, 111	0. 9853	0.023	0.068
diisobutyl adipate	DiBA	17.1	129, 185, 111	0.9955	0.008	0.027
di(2-ethylhexyl) adipate	DEHA	30.9	129, 112, 147	0.9865	0.013	0.037
di(2-ethylhexyl) terephthalate	DEHT	37.5	149, 112, 261	0.9883	0.070	0.233
di(2-ethylhexyl) sebacate	DEHS	38.2	185, 149, 112	0.9933	0.018	0.053

Underlined ions were considered for quantitative analysis.

Table S6. List of the investigated bisphenols with Retention Time and Monitored ions (m/z), linearity, LOD, LOQ.

Compound	t_R (min)	Monitored ions (*m/z*)	Linear range (μg/ kg)	r^2	LOD (μg /Kg)	LOQ (μg /Kg)
4,4′-Sulfonyldiphenol (BPS)	10.7	107.9, 92.0, 156.0	1-250	0.9993	0.30	1
4,4′-Methylenediphenol (BPF)	14.6	93.1, 105.1	1-250	0.9911	0.45	1.5
1,1-Bis(4-hydroxyphenyl) ethane (BPE)	15.9	198.0, 194.9, 176.9	1-250	0.9931	0.30	1
4,4'-(propan-2,2-diyl) diphenol (BPA)	16.9	212.1, 133.0, 211,1	1-250	0.9907	0.45	1.5
4-[2-(4-hydroxyphenyl) butan-2-yl] phenol (BPB)	19.1	212.0, 211.0	1-250	0.9944	0.30	1
2,2-Bis(4-hydroxyphenyl) exafluoropropane (BPAF)	19.7	265.0, 177,0 69,0	1-250	0.9995	0.30	1
1,1-Bis(4-hydroxyphenyl)-1-phenyl-ethane (BPAP)	19.9	274.1, 273.1, 211,0	1-250	0.9954	0.45	1.5
1,1-Bis(4-hydroxyphenyl)-cyclohexane (BPZ)	20.5	145.0, 173.1, 222.9	1-250	0.9984	0.45	1.5
1,4-Bis(2-(4-hydroxyphenyl)-2-propyl)benzene (BPP)	24.1	330.1, 133.1, 314,9	1-250	0.9992	0.45	1.5

Underlined ions were considered for quantitative analysis.

Table S7. Estimated Daily Intake (μg/kg$_{bw}$/day), Hazard Index and Tolerable Daily Intake (μg/kg$_{bw}$/day) for plasticizers and bisphenols from Provola samples categorized by diet and period.

	DMA	DMP	DEP	DPrP	DiBP	DBP	DEHA	DEHP	DEHT	Sum of DBP and DEHP	*BPAF*	*BPS*
**TDI**			5000			10	300	50		50		
**EDI**												
**CTR**												
March	3.5×10^{-3}	3.2×10^{-3}	4.6×10^{-3}	2.4×10^{-3}	1.2×10^{-2}	4.6×10^{-3}	7.4×10^{-2}	1.6×10^{-2}	3.9×10^{-2}	2.0×10^{-2}	1.5×10^{-3}	1.3×10^{-3}
April	3.9×10^{-3}	3.0×10^{-3}	4.0×10^{-3}	2.0×10^{-3}	1.1×10^{-2}	4.3×10^{-3}	3.8×10^{-2}	1.4×10^{-2}	4.5×10^{-2}	1.8×10^{-2}	1.7×10^{-3}	1.6×10^{-3}
May	3.7×10^{-3}	3.2×10^{-3}	3.9×10^{-3}	2.5×10^{-3}	1.2×10^{-2}	4.7×10^{-3}	3.5×10^{-2}	1.5×10^{-2}	3.9×10^{-2}	1.9×10^{-2}	2.1×10^{-3}	1.6×10^{-3}
June	5.3×10^{-3}	3.2×10^{-3}	5.3×10^{-3}	2.3×10^{-3}	9.1×10^{-3}	4.9×10^{-3}	6.8×10^{-2}	1.5×10^{-2}	4.1×10^{-2}	2.0×10^{-2}	2.0×10^{-3}	1.8×10^{-3}
July	3.6×10^{-3}	3.8×10^{-3}	5.3×10^{-3}	2.1×10^{-3}	1.3×10^{-2}	6.8×10^{-3}	7.1×10^{-2}	1.1×10^{-2}	9.3×10^{-2}	1.6×10^{-2}	2.1×10^{-3}	1.9×10^{-3}
**BIO**												
March	3.4×10^{-3}	3.2×10^{-3}	4.6×10^{-3}	2.4×10^{-3}	1.2×10^{-2}	4.7×10^{-3}	7.4×10^{-2}	1.6×10^{-2}	3.9×10^{-2}	2.0×10^{-2}	1.7×10^{-3}	1.5×10^{-3}
April	3.9×10^{-3}	3.1×10^{-3}	4.3×10^{-3}	2.0×10^{-3}	1.1×10^{-2}	4.4×10^{-3}	3.8×10^{-2}	1.4×10^{-2}	4.5×10^{-2}	1.9×10^{-2}	1.9×10^{-3}	1.5×10^{-3}
May	3.7×10^{-3}	3.1×10^{-3}	3.9×10^{-3}	2.4×10^{-3}	1.2×10^{-2}	4.6×10^{-3}	3.4×10^{-2}	1.5×10^{-2}	4.0×10^{-2}	1.9×10^{-2}	1.9×10^{-3}	1.5×10^{-3}
June	5.3×10^{-3}	3.1×10^{-3}	5.3×10^{-3}	2.3×10^{-3}	9.1×10^{-3}	4.8×10^{-3}	6.7×10^{-2}	1.5×10^{-2}	4.1×10^{-2}	2.0×10^{-2}	1.8×10^{-3}	1.6×10^{-3}
July	3.2×10^{-3}	3.8×10^{-3}	5.3×10^{-3}	2.2×10^{-3}	1.2×10^{-2}	6.7×10^{-3}	7.1×10^{-2}	1.1×10^{-2}	8.8×10^{-2}	1.7×10^{-2}	2.0×10^{-3}	1.8×10^{-3}
**HQ**												
**CTR**												
March			9.1×10^{-7}			4.6×10^{-4}	2.5×10^{-4}	3.2×10^{-4}		4.1×10^{-4}		
April			8.1×10^{-7}			4.3×10^{-4}	1.3×10^{-4}	2.9×10^{-4}		3.7×10^{-4}		
May			7.8×10^{-7}			4.7×10^{-4}	1.2×10^{-4}	3.0×10^{-4}		3.8×10^{-4}		
June			1.1×10^{-6}			4.9×10^{-4}	2.3×10^{-4}	3.0×10^{-4}		4.1×10^{-4}		
July			1.1×10^{-6}			6.8×10^{-4}	2.4×10^{-4}	2.1×10^{-4}		3.2×10^{-4}		
**BIO**												
March			9.1×10^{-7}			4.7×10^{-4}	2.5×10^{-4}	3.2×10^{-4}		4.1×10^{-4}		
April			8.6×10^{-7}			4.4×10^{-4}	1.3×10^{-4}	2.9×10^{-4}		3.7×10^{-4}		
May			7.8×10^{-7}			4.6×10^{-4}	1.1×10^{-4}	3.0×10^{-4}		3.8×10^{-4}		
June			1.1×10^{-6}			4.8×10^{-4}	2.2×10^{-4}	3.0×10^{-4}		4.0×10^{-4}		
July			1.1×10^{-6}			6.7×10^{-4}	2.4×10^{-4}	2.3×10^{-4}		3.3×10^{-4}		

n.a., not available.
